# Statistical image analysis and escort histograms in characterization of articular cartilage repair in a skeleton animal model

**DOI:** 10.1371/journal.pone.0252505

**Published:** 2021-06-18

**Authors:** Ryszard Tomaszewski, Jerzy Dajka

**Affiliations:** 1 Department of Pediatric Traumatology and Orthopedy, Upper Silesian Child Centre in Katowice, Katowice, Poland; 2 Faculty of Science and Technology, Institute of Biomedical Engineering, University of Silesia in Katowice, Katowice, Poland; 3 Faculty of Science and Technology, Institute of Physics, University of Silesia in Katowice, Katowice, Poland; 4 Silesian Center for Education and Interdisciplinary Research, University of Silesia in Katowice, Chorzów, Poland; University of Life Sciences in Lublin, POLAND

## Abstract

Statistical image analysis of an ensemble of digital images of histological samples is performed as an auxiliary investigation a result of the recently proposed method of articular cartilage repair utilizing growth plate chondrocytes in a skeleton animal model. A fixed–shift model of maximal likelihood estimates of image histograms applied for monochromatic (grayscale) images or their RGB components confirms the statistically significant effect of the previously proposed medical treatment. The type of staining used to prepare images of histological samples is related to the visibility of the effectiveness of medical treatment. Hellinger distance of escort distributions for maximal likelihood estimates of image histograms of medically treated and control samples is investigated to identify grayscale (or RGB) intensities responsible for statistically significant difference of the estimates. A difference of Shannon entropy quantifying informational content of the histograms allows one to identify staining and image colors which are most suitable to visualize cluster formation typical for articular cartilage repair processes.

## Introduction

Examination of digital images serves as a powerful diagnostic tool in medical practice and research. There are highly sophisticated methods of visualising samples being either non–invasive (with magnetic resonance imaging as an example) or more invasive when experienced pathologists microscopically examine surgically acquired histological preparations. The common practice of comparison of images of tissues before and after medical treatment to confirm or discover a medical effect suffers from well–known drawbacks due to e.g. both inter– and intra–image variations [1] or a problem of examiner reliability [2] which need to be taken into account. Statistical methods of an image analysis can cure at least some of those problems and serve, as we show in this paper, as an auxiliary tool for supplementary verification for orthopaedic medical findings or gaining a complementary view revealing new features.

Due to its specific structure and highly specialised function the treatment of damage to articular cartilage is a major clinical problem. In response, regenerative medicine develops new techniques and methods for cartilage defect treatments involving cell therapy when isolated and specially cultured cells become injected into the joint or via tissue engineering techniques and can be implanted on special substrates. The authors of a recent medical study [3], using histological animal samples (immature pigs), compare the effectiveness of two types of treatment of cartilage damage repair: *(i)* bone marrow stimulation (chosen as a reference) and *(ii)* autologous growth plate chondrocytes therapy. The results showed that addition of immature chondrocytes provides new possibilities for articular cartilage treatment. Moreover, the presence of chondrocyte clusters formed during cartilage regeneration was emphasised in the paper [3] as an interesting and not fully explained manifestation of the cartilage response to the stress factor. Both the medical importance of the problem of damage to articular cartilage and the existence of open, still unsolved problems justifies using a statistical approach complementing the recent investigations [3].

In this paper we analyze optical images of the histological preparations used in the above mentioned study [3] using statistical methods based on histograms related to the digital images. Image histograms contain useful information which can be extracted and utilized to verify, support or question the effectiveness of medical treatment [4, 5]. Having two sets of two–dimensional images of medically treated and control samples with a single grayscale intensity (for monochromatic pictures) or a list of three intensities (for RGB colored pictures) attached to each pixel of the image (forming a two–dimensional array), one infers how many times a given intensity level appears in the array of pixels. This information allows one to construct a histogram associated with a given image. Further statistical modelling, however, depends on a set of (crucial) assumptions such as proper identification of the general population the sample comes from or to what extent the probability distribution may differ from sample to sample. Here we compare selected descriptors of histograms estimated under assumptions satisfied by a *fixed shift model* [1] i.e. we assume that individual images may differ in nothing but fixed (and random) ‘intensity shift’. Such an approach is placed somewhere in between the restrictive case of identically distributed intensities of individual histograms and the ‘liberal one’ where all the distributions are different. More precisely, for the model we apply one assumes images belonging to the same general populations but with a random and image–dependent variation allowed. Statistical significance in difference of estimated descriptors requires hypothesis testing [6] either parametric or non–parameteric (robust) depending on the type of modelling of the image distribution [1] and verification of requirements for using parametric models [6]. Improper choice is a source of doubtful conclusions [7, 8]. For a non–parametric comparison we apply the Kolmogorov-Smirnov test [6]. Its application to statistical image comparison suffers from known [4] limitations: as a two–sided test one cannot easily infer significance of statistical difference of certain quite natural image descriptors e.g. to infer a relation of overall darkness of images belonging to compared sets. Using the Kolmogorov–Smirnov test one also rejects its null hypothesis for histograms differing only in a single grayscale intensity [4, 6]. It motivates the application of more elaborate methods such as a fixed shift model [1] employed also here. In this paper we investigate Hellinger distance [9] between fixed–shift model estimated histograms. One method for a better scanning histograms (probability distributions) is to analyse one–parameter families of *escort distributions* (escort histograms) [10, 11] studied in a broad range of problems of superstatistics [12] starting from evolutionary games [13] via statistics of earthquakes [14] up to mathematical aspects [15–17] and its relation to celebrated Tsallis entropy [13]. Escort histograms allow one to enhance the contribution of certain groups of intensities *g* characterized e.g. by large values of *p*_*g*_. The Hellinger distance between escort histograms of medically treated and control groups becomes maximised for pairs of escort histograms which are most distant from each other and, as a result, optimally visualize an effect of medical treatment.

It is a common practice to apply entropy (or in general various entropies) to quantify informational content of images [18–20]. Here, following these approaches, we compare the Shannon entropy of an escort histograms of medically treated and control groups. As the Shannon entropy quantifies an amount of information content of a probability distribution we expect entropy increase to indicate formation of non–uniform regions in a histologic sample related to formation of *clusters* which are characteristic for articular cartilage repair process. The presence of chondrocytes with cytoplasmic processes, increased volume and clustering suggests important early changes to their phenotype [21]. Clusters are composed of at least three chondrocytes [21]. They most commonly develop in degenerative changes but may also accompany immobilization or joint trauma [22, 23]. However, Lotz et al. [24] associate cluster formation with a response to changing mechanical conditions and regeneration within the articular cartilage. There are many studies of the shape of chondrocytes and their metabolism and the formation of clusters is still being investigated [21]. We show that Shannon entropy for image histograms indicates medical treatment and cluster formation and is associated with a type of staining used and an RGB component of a digital microscopic image.

The paper is organized as follows: after reviewing applied methods and constructing fixed–shift maximal likelihood estimates (MLE) for histograms of medically treated and control groups and verifying their statistically significant difference we juxtapose corresponding cumulative probability distributions for different types of staining of histologic samples both the monochromatic grayscale and the RGB image components. Next, for MLE histograms, we construct their *escort histograms* [10]. We present and analyse Hellinger distance between escort histograms of medically treated and control groups for different staining grayscale or RGB–component. We identify intensities influencing statistical difference of images. Further, for the same pairs of escort histograms, we investigate the difference in Shannon entropy large values of which indicate formation of clusters due to an applied articular cartilage repair process. Finally we discuss and summarize our results.

## Methods

**Fig 1:**
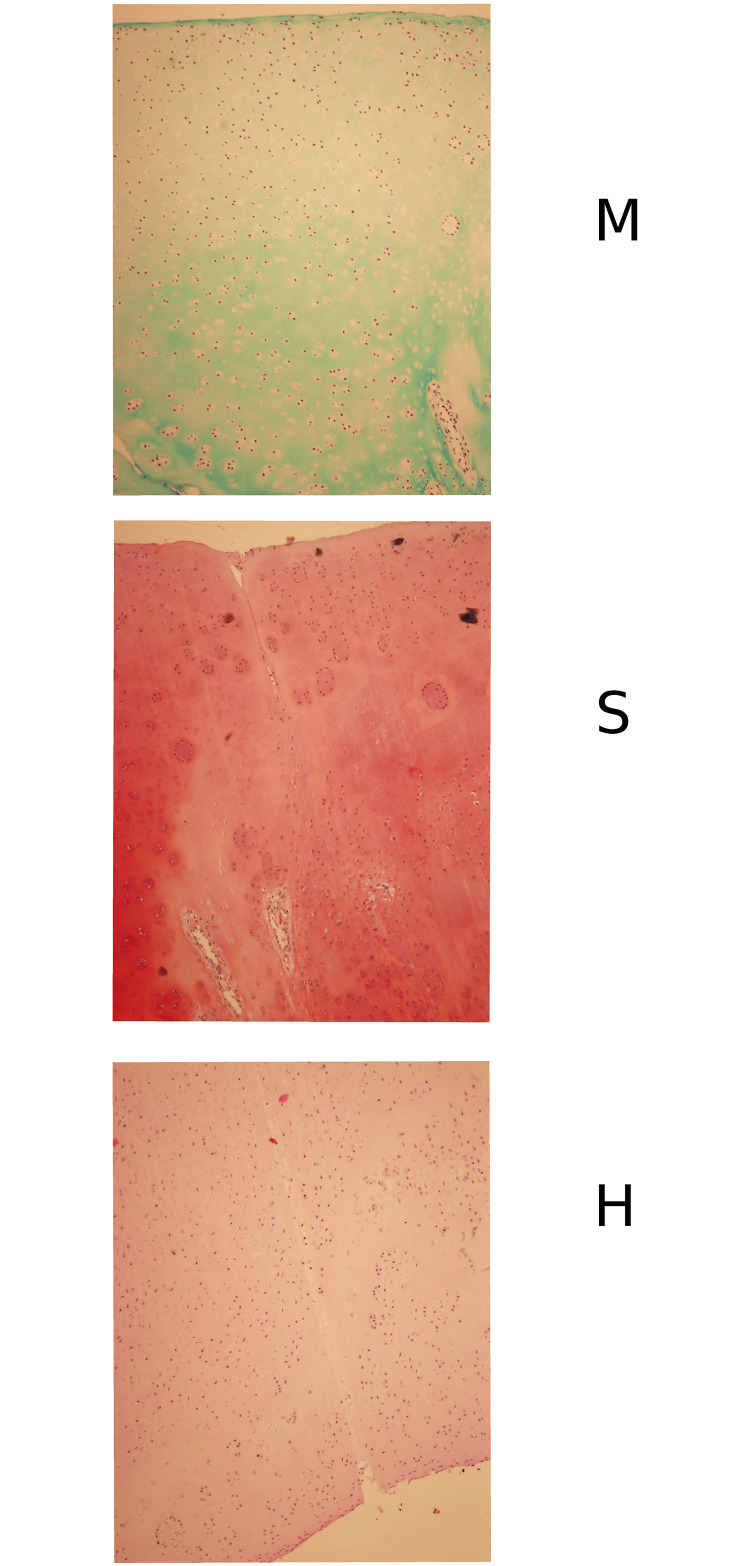
Examples of microscopic images of histological samples. *H*: stained with haematoxylin and eosin, *S*: stained with Safranin and *M*: stained with Masson’s trichrome.

We analyze optical images of the histological preparations which (for an evaluation conducted by pathologists reported in Ref. [3]) were stained with either *(i)* haematoxylin and eosin (abbreviated as ‘H’ below) or *(ii)* Safranin O (abbreviated as ‘S’ below) or *(iii)* Masson’s trichrome (abbreviated as ‘M’ below) resulting in images of different colour, cf. Fig 1 where we present three examples. Any digital image is nothing but a matrix of scalars *g* = 0, …, 255 (for monochromatic images) or, for color images, a matrix of three–element lists of scalars [*g*_*R*_, *g*_*G*_, *g*_*B*_] denoting (grayscale) intensity of red, green and blue (RGB) color respectively. A monochromatic image can be described by a histogram *p*_*g*_, *g* = 0, …, 255 where *p*_*g*_ denotes the probability of finding a pixel with an grayscale intensity *g*. Similarly, with a colored image one associates a set of three histograms of monochromatic images corresponding to the three colors of the RGB classification. Note that although members of such a triple are usually not independent, cf. Ref. [5], in this work we do not discuss the effect of color–color correlations. Let us note that not only does a different type of staining result in different color of a sample but also the intensity of the applied staining can vary from sample to sample. This justifies our choice of the *fixed–shift model* [1] for maximal likelihood estimation of histograms of medically treated and control groups to be later statistically compared. Moreover, if the histograms are different and the difference is known to be statistically significant, one can investigate the origin of the difference and verify which among the intensities *g* = 0, …, 255 non–trivially contribute to the difference.

Construction of histograms is done using Python toolbox scikit-image which provides algorithms particularly devoted to image processing and the Python module collections. Statistical analysis of histograms is conducted with NumPy and SciPy—the two fundamental Python packages suitable for scientific computing and, in particular, with scipy.stats for the Kolmogorov–Smirnov comparison test. The procedure of construction and analysis of histograms applied in our work consists of the following steps which are linear and can be summarized as follows: *(i)* selection of type of staining: H,S,M; *(ii)* reading jpg images; *(iii)* color selection: R,G,B or gray; *(iv)* counting grayscale intensities; *(v)* MLE estimation of histogram; *(vi)* Kolmogorov–Smirnov comparison for medically treated and control groups; *(vii)* construction of escort histograms; *(viii)* analysis of the Hellinger distance and Shannon entropy. Let us emphasise that all but the last two steps are typical for statistical image analysis [1].

The *fixed–shift model* [1] is a first approximation for statistical image analysis taking into account intensity of staining varying from image to image. It allows one to find the maximal likelihood estimate (MLE) for histogram {*p*_*g*_} of a set of unstructured images belonging to the same population. It is assumed that individual images result from sampling of a single population and their histograms differ only in a fixed (image–dependent) shift. This model has been successfully applied [1] for hypoxia BOLD MRI rat brain data [25]. Here, instead of MRI images, we consider three groups (differing in the type of applied staining: H, S or M) of optical 1024 × 1360 dimensional images presenting animal histological samples [3]. Each of three groups splits into two classes: the first, serving as a control group, where the treatment was limited to scaffold only, the second, where the novel approach—proposed in Ref. [3] and utilizing growth plate chondrocytes cells—was applied. We have at our disposal 8+8 images for S–staining and 7+7 images for M– and H–staining.

After the logit transformation [1] (limited to non–zero *p*_*g*_ ≠ 0 in an *i*–th image in an ensemble):

$$\begin{array}{c} \rho_{g}=\text{ln}\;p_{g}-\text{ln}\;p_{0} \end{array}$$ (1)

assuming a possible image-*i*-dependent fixed shift:

$$\begin{array}{c} \rho_{g}\to \rho_{g}+b_{i} \end{array}$$ (2)

calculating MLE one arrives at an estimate

$$\begin{array}{c} \rho_{g}=\text{ln}\;(\frac{d_{g\neq 0}}{N_{T}-d_{0}}), \end{array}$$ (3)

where, as shown in Ref. [1], *N*_*T*_ is a total number of pixels in an ensemble, *d*_*g*_ is the frequency of a grayscale *g*–level in an ensemble of images. To qualify an effect of medical treatment, i.e. to show a statistically non-trivial impact of growth plate chondrocyte cells (versus scaffold), we utilize the Kolmogorov–Smirnov test for comparing MLE histograms [4] of images with or without cells with a significance set at p-value ≤0.05. With histograms {*p*_*g*_} one associates a cumulative distribution *F*_*g*_ = ∑_*h*<*g*_ *p*_*h*_. A horizontal relative shift of two cumulative distributions of digital images qualifies one of the compared histograms as being darker than the other.

To quantify dissimilarity of images we utilize Hellinger distance [9] between the histograms {*p*}, {*q*}:

$$\begin{array}{c} H^{2}\left(\left\{p\right\},\left\{q\right\}\right)=\frac{1}{\sqrt{2}}\sum_{g}{\left(\sqrt{p_{g}}-\sqrt{q_{g}}\right)}^{2} \end{array}$$ (4)

Let us notice that Kullback–Leibler information divergence [10] is another candidate serving as a tool for histograms comparison which, however, is not symmetric lacking an interpretation as a geometric distance. Two different histograms {*p*} and {*q*} are separated in the Hellinger distance provided that their individual probabilities *p*_*g*_ ∈ {*p*_*g*_} and *q*_*g*_ ∈ {*q*_*g*_} differ. To identify intensities *g* which most significantly contribute to the Hellinger distance, we associate with a given histogram {*p*_*g*_} its related *escort distribution* [10] (escort histogram) {*P*_*g*_(*β*)} reading

$$\begin{array}{ccc} P_{g}\left(\beta \right) & = & p_{g}^{\beta }/\sum_{p_{h}\neq 0}p_{h}^{\beta },\;\;\;\beta \in R \end{array}$$ (5)

where an applied parameter *β* allows the histogram {*p*_*g*_} to be ‘scanned’ to enhance the impact of large (resp. small) *p*_*g*_ ∈ {*p*_*g*_} with an increasing (resp. decreasing) value of *β* in {*P*_*g*_(*β*)}.

Deputizing a histogram for an original image results in unavoidable loss of information: there are many images having in principle the same histogram. A partial remedy for this drawback is to quantify information carried by a histogram (or of an associated escort histogram) via its Shannon entropy [1] defined as [10]

$$\begin{array}{cc} S(\left\{P_{i}\left(\beta \right\}\right) & =-\sum_{\left\{P_{i}\left(\beta \right)\right\}}P_{i}\left(\beta \right)\;\text{ln}\;P_{i}\left(\beta \right) \end{array}$$ (6)

Let us emphasise that motivated by information–theoretic rather than thermodynamic properties we apply Shannon entropy rather than the more general Renyi construction [10, 11] already containing a parameter *β* in its definition. Note that lower Shannon entropy, indicating existence of high probabilities of a smaller number of intensity values, serves as a hallmark of potentially existing uniform regions whereas a sample of large entropy may contain clusters induced by medical treatment [3].

The MLE [1] described above is performed for a set of histograms constructed for individual monochromatic images either monochromatic i.e. black-and-white (gray) or constricted by extracting the R– (red), G– (green) or (blue) B–component from an RGB color image. Hence a single image leads to four empiric histograms characterized by its color component which is gray, red, green or blue. Moreover, the images (and each of their histograms) are characterized by the type of applied staining (S,M,H) and the medical treatment used i.e. with or without cells added to enhance the repair process.

## Results

**Fig 2:**
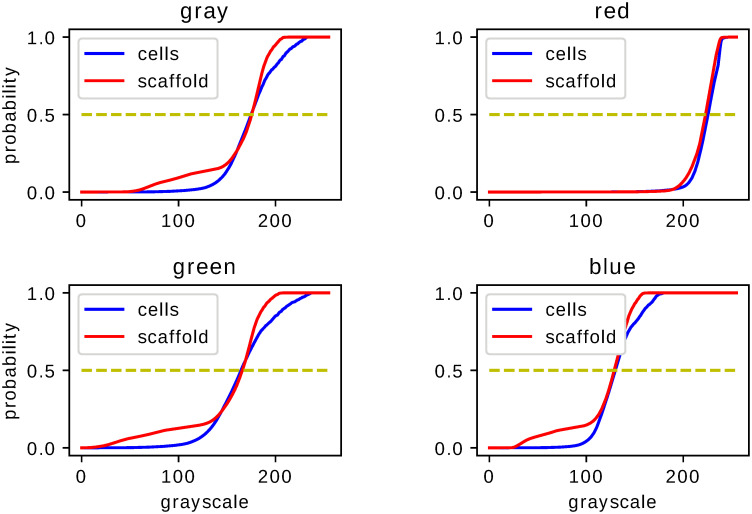
Cumulative histograms for H–stained samples. Cumulative distributions of fixed–shift MLE Eq (3) of ensemble of images of H–stained samples. The two graphs juxtaposed in each figure correspond to the samples with or without cell–based repair applied. Each figure corresponds to image histograms for different colors: gray (for grayscale monochromatic image), red, green and blue for the RGB–classification.

**Fig 3:**
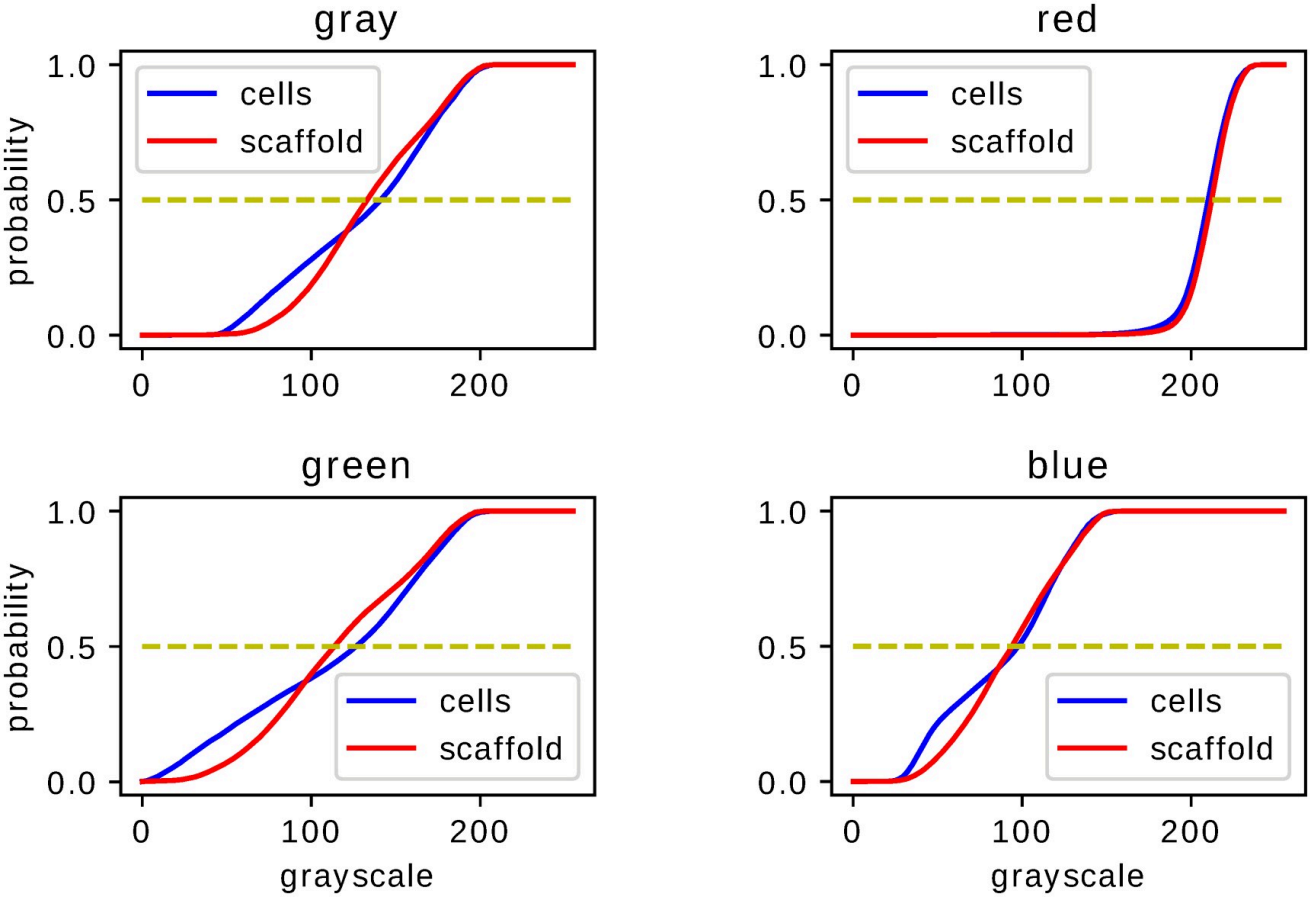
Cumulative histograms for S–stained samples. The same as in Fig 2 but for S–stained images.

**Fig 4:**
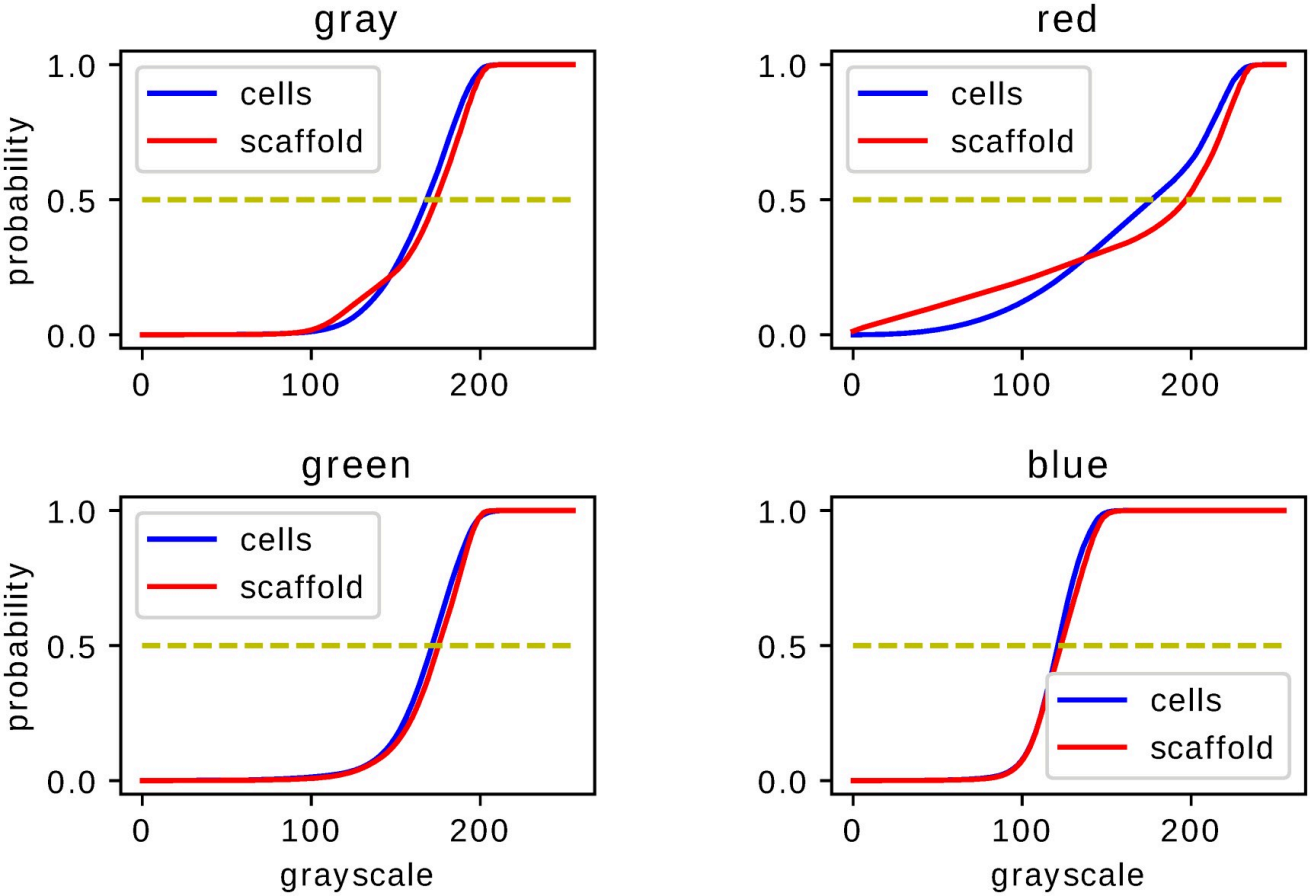
Cumulative histograms for M–stained samples. The same as in Figs 2 and 3 but for M–stained images.

**Fig 5:**
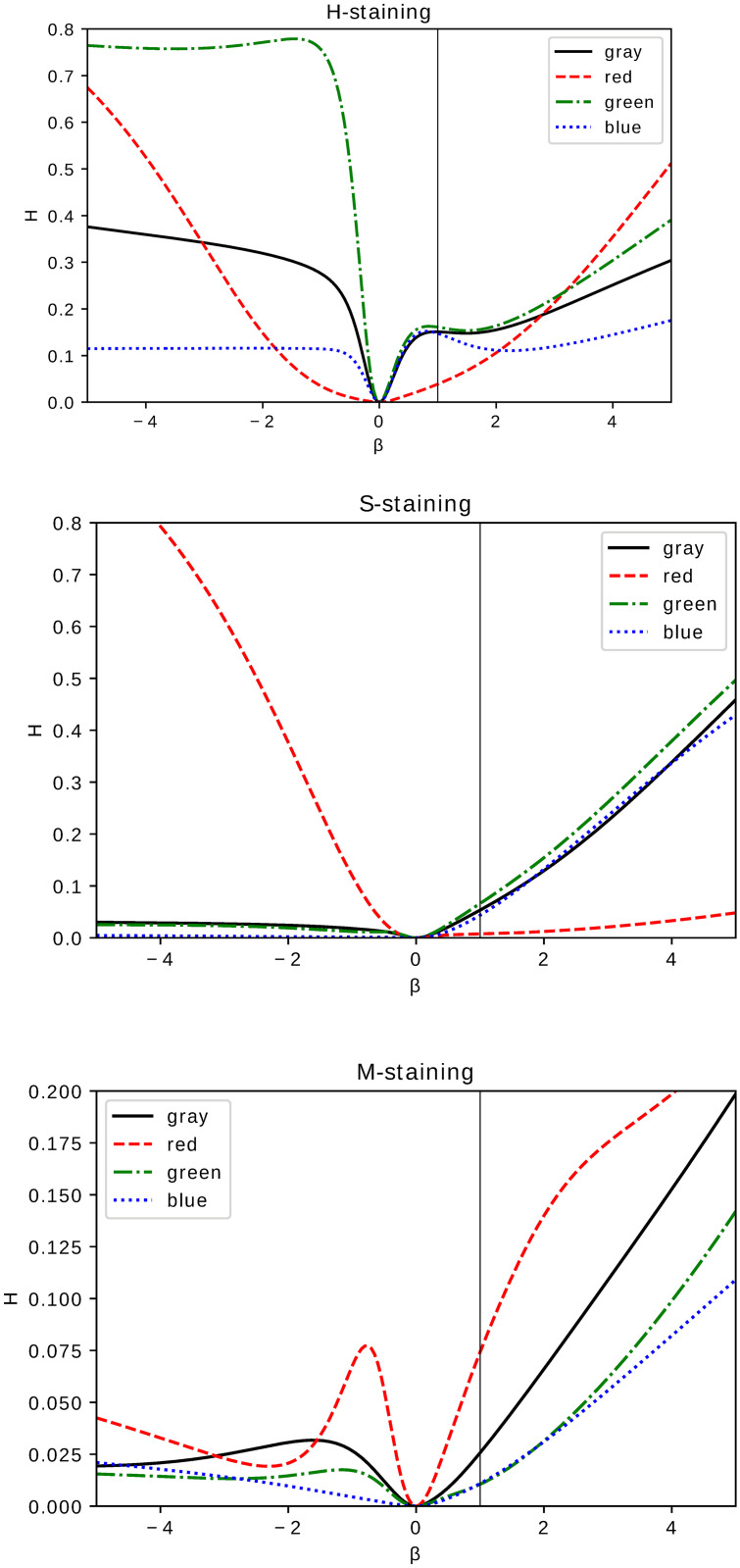
Hellinger distance Eq (4) between escort histograms of medically treated and control groups as a function of *β*. The panels correspond to different staining: H–staining (upper panel), S–staining (middle) and M–staining (bottom panel). The lines in each panel correspond to a pair of histograms of images in a given color: gray for grayscale monochromatic, red, blue and green for RGB–components of digital images.

Statistical comparison of the fixed–shift model MLE histograms of histological images shows, upon Kolmogorov–Smirnov test, significant difference resulting from medical treatment proposed in Ref. [3]. This property supports claims of Ref. [3] concerning the effectiveness of the proposed treatment. The analysis performed for images of different color (monochromatic or RGB) and different type of staining (H,S,M) leads to a p-value never exceeding 0.05. In Figs 2–4 we present the cumulative distribution of MLE histograms of various colors of images of samples stained by H, S and M respectively. There are features of the presented set of cumulative distribution which need to be emphasised. Except H–staining cf. Fig 2 there is no common tendency allowing one to claim that medical treatment leads to darker or lighter images in comparison with a scaffold filling of the injury. Moreover, inspection of auxiliary yellow lines drawn at 1/2 in Figs 2–4 indicates similarity of median values of the histograms intensities *g*. As the mean values of intensities, not presented here, are also very close they can hardly serve as a criterion to distinguish medical treatment and control groups. In particular, let us note that except for M–staining the red–color histograms are very close to each other which indicates their limited applicability for analysis of histological samples with H– and S–staining applied. MLE histograms for both groups—medically treated and control—although (upon the Kolmogorov–Smirnov test results) statistically different, require further analysis to extract the origin of their difference. For MLE histograms we analyse escort distributions Eq (5) allowing, via changing *β*, the role played by a particular histogram’s probabilities of comparable amplitude to be enhanced. MLE histograms or cumulative distributions, despite their statistical difference confirmed by the Kolmogorov–Smirnov test, are seemingly similar. One can suspect that significant differences occur for intensities *g* of small probability and hence are at first glance difficult to observe. One possible way to overcome this difficulty is to utilize, instead of MLE histograms, their escort distributions Eq (5) and, by changing the parameter *β*, enhance the differences otherwise hardy visible. In Fig 5 we present the Hellinger distance between escort distributions of MLE histograms corresponding to medically treated and control groups as a function of *β*. As for *β* = 0 every escort distribution is uniform, the Hellinger distance vanishes. For *β* = 1, indicated by a vertical solid line in each panel of Fig 5, the Hellinger distance is the distance of the original MLE histograms. Let us note significant dependence on both the type of staining (H,S or M) and a color chosen to build the histogram. There are three qualitatively different behaviours of the distance between escort histograms as a function of *β* presented in Fig 5. The first *(i)*, is characterized by monotonic growth of a distance as function of *β* indicating influential role of pixel ‘tail intensities’ *g* whose probabilities are small or large in comparison to typical or ‘central’. The second *(ii)*, has an almost flat *β*–characteristics of the Hellinger distance indicating no particular role played by any particular subset of intensities in the histogram, no matter what their probabilities are. It allows one to infer that in such a case differences in probabilities of all *g* are ‘equally responsible’ for the statistical difference of MLE histograms indicated by the Kolmogorov–Smirnov test. The third *(iii)*, has a *β*–characteristics which fall neither in the first nor in the second class but are non–monotonic. The Hellinger distance as a function of *β* exhibits (either for positive or negative *β*) maximum indicating escort histogram ‘optimally’ presenting an effect of the applied medical treatment. It if particular interest if the optimum occurs for *β* = 1 i.e. for the original MLE histograms indicating conditions favouring direct image comparison.

**Fig 6:**
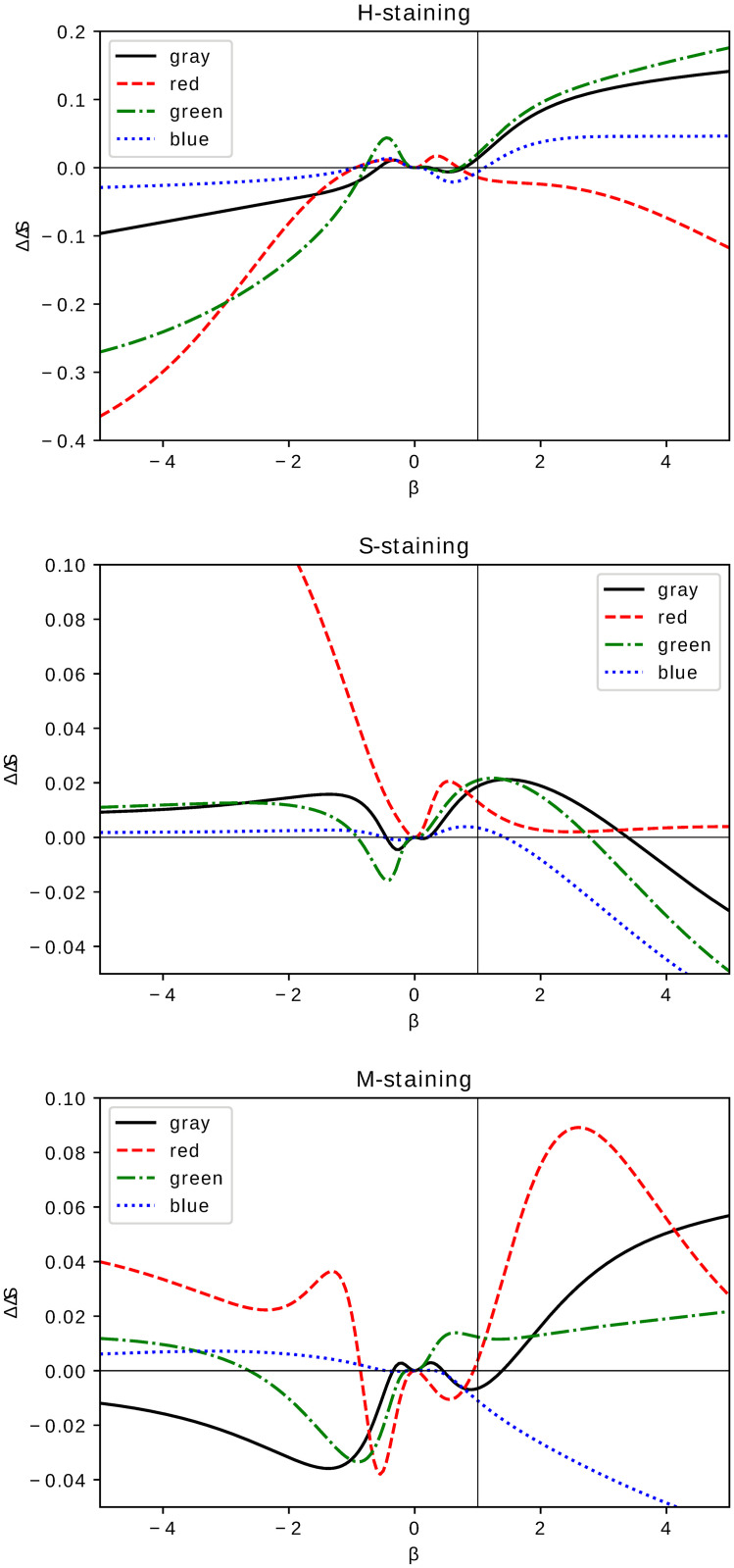
Entropy difference. Shannon entropy Eq (7) difference between escort histograms of medically treated and control groups as a function of *β*. The panels correspond to different staining: H–staining (upper panel), S–staining (middle) and M–staining (bottom panel). The lines in each panel correspond to a pair of histograms of images in a given color: gray for grayscale monochromatic, red, blue and green for RGB–components of digital images.

Informational content of escort histograms, quantified by a difference of entropy Eq (6)

$$\begin{array}{ccc} \Delta S & = & S(cells)-S(control) \end{array}$$ (7)

where *S*(*cells*) (resp. *S*(*control*)) are Shannon entropies [10] of escort histograms of images of samples medically treated by with growth plate chondrocytes or scaffold–treated control group, is presented in Fig 6 for different staining applied. Using entropy decrease as a quantifier of formation of uniform (with a dominant grayscale intensity) regions makes it possible to identify values of *β* which, for a given staining, are most favourable for detecting formation of clusters [3] due to the applied medical treatment. For an H–staining, setting *β* > 1 leads to Δ*S* > 0 which allows (for all colors except red) ‘visibility’ of cluster formation to be enhanced as it is presented in the upper panel of Fig 6. The entropy difference Δ*S* for S–staining presented in the central panel of Fig 6 indicates an inverse tendency: for most colors (partially except green) a range of *β* leading to Δ*S* > 0 is bounded from above by a small but positive value *β* < 4. However, small value of Δ*S* (with the exception of a red color histogram) makes S-staining unlikely for studying potential cluster formation. Entropy difference Δ*S* for the M–stained samples is presented in the bottom panel of Fig 6. Note less regular behaviour being strongly color–dependent. For the grayscale one observes similar behaviour as it was for H–staining in the top panel of Fig 6 with Δ*S* < 0 (Δ*S* > 0) for *β* < 1 (resp. *β* > 2). However, the entropy difference of the red RBG–component of images’ histogram, except the central region of −2 < *β* < 2, is positive indicating potential utility for studying entropy growth of histological microscopic samples due to medical treatment.

## Discussion

Statistical analysis of histograms serving as an auxiliary tool for image comparison has certain advantages effective use of which depends on various features and parameters. Our investigations concern colored images of histological samples stained by three types of dyes. Provided that statistical difference inferred from the Kolmogorov–Smirnov test [4] indicates successful medical treatment [3] of an injury we recognized conditions which lead to the most significant and visible difference. Our results cf. Figs 2–4 indicate that the features which need to be taken into account to improve effectiveness of statistical image comparison are both the color and the staining. According to the fixed–shift model [1] applied in our work Eq (3) the ‘most visible’ difference in cumulative distributions of the red component of an RGB image occurs for M–stained samples i.e. if one is going to limit to a ‘single glance’ inspection of a red component of images, it can be most effectively done for M–stained samples since for both H– and S–staining cumulative distribution of red component of images are hardly distinguishable despite their formal statistical difference indicated by the Kolmogorov–Smirnov test. This is due to close values of typical characteristics such as mean or median and lack of tendency to associate a darker or lighter image with a medically treated sample. The properties exemplified above encourage one to search for effective tools which allow one to enhance the difference making it more significant.

Escort histograms [10] Eq (5) which we consider seem to fill at least partially this requirement serving for a supplementary viewpoint. They allow—by a proper choice of a parameter *β* in Eq (5)—one to scan the gray–scale intensity distribution to recognize groups of intensities (of a large or small probability) leading to large values of Hellinger distance Eq (4) of escort histograms Eq (5). Such a strategy, despite its effectiveness, needs to be applied in relation to a chosen RGB or gray (for monochromatic images) component of images leading to an estimation of a fixed–shift histogram. In particular, this strategy becomes effective if the Hellinger distance either significantly increases or becomes maximised in a well–defined tailored range of *β* in Eq (5) corresponding e.g. to large or small probabilities in a MLE image histogram. On the other hand, a Hellinger distance which is almost flat indicates MLE escort histograms whose difference cannot be associated with a particular group of probabilities and is ‘uniformly’ distributed among all image intensities. The Hellinger distance of escort histograms effectively quantifies image distinctiveness under conditions which are both color– and staining–dependent as presented in Fig 5 and—to show its effectiveness—one must limit neither to a particular color nor staining.

Shannon entropy Eq (6) quantifies information content of MLE histograms. Its interpretation is well known [10, 11] and utilized in a wide spectrum of problems from thermodynamics to chaos theory and communication [26]. It can indicate formation of clusters [21–24] known to be characteristic for an injury repair process. Extreme or large values of Δ*S* Eq (7) in Fig 6 allow one to identify a tailored range of *β* in Eq (5) making a particular escort histograms best applicable to indicate cluster formation. Again, as it was for Hellinger distance, Shannon entropy can serve as a auxiliary tool to infer effectiveness of medical treatment from a digital image of histological samples. In particular, using Shannon entropy of escort histograms we confirm formation of clusters [21–24] already observed in Ref [3] and indicate cf. Fig 6 for a given staining of a histological sample a color component of an RGB image most suitable for its verification. Image analysis focused on image histograms instead of full images is an attractive alternative to standard image descriptors such as a mean or variance. Histograms allow for a *global* description of an object and in particular they allow ones to distinguish and take into account global (unwanted) features and noises. We presented a particular application of this property using the fixed shift model for an ensemble of histological samples globally perturbed by applied staining. One can consider a histogram of a digital image as a particular descriptor of a very rich informational content which, last but not least, is easy to handle if one tries to avoid computationally expensive techniques necessary for large digital images being often resource intensive. The method of using escort histograms proposed in this work belong to a class of auxiliary tools suitable for correction of image quality such as image equalization. Scanning the one–parameter family of escort histograms one can recognize the global features which significantly affect a particular descriptor such as entropy of Hellinger distance studied here. Let us notice that the values of *β* optimally enhancing visibility of a given feature depend on the chosen descriptor. A potential replacement of the Hellinger distance by related metrics such as the Bhattacharyya coefficient or even Euclidean or Manhattan distance does not lead to qualitatively different results. However, histogram users should remember that there is a price to pay: passing from an original image to its histogram results in unavoidable information loss as there are many distinct images having statistically the same histogram. It is not the only limitation of the proposed method of medical image treatment: concentrating on global features results in an often unsatisfactory insight into details of an image structure. In particular, using escort histograms allows one to confirm formation of chondrocyte clusters in the repair processes but says nothing about their shape and size. Moreover, being limited to one–dimensional histograms (grayscale intensity) we neglect inter–color correlations which are known to be a significant feature of medical images [5] and which, together with investigation of a spatial structure of digital images and shape analysis [1] of clusters formed in articular cartilage repair processes, are natural directions of future investigations based on the results of the experiment [3].

## Conclusion

Statistical image analysis [1], used as an auxiliary tool, effectively supplements ‘standard’ medical analysis of histological samples [4, 5] usually done by experienced pathologists [3]. It is advantageous since its use allows one to avoid the common problem of examiner reliability [2] and it can can easily be ‘automatised’ with a relatively small numerical effort. On the other hand, passing from a full image into its histogram results in information loss which needs and to be compensated. In this work we described how statistical image analysis can supplement medical investigation of the highly important orthopaedic problem of femoral articular cartilage injury studied for an animal model. Our approach not only allows one to confirm claims formulated in Ref. [3] but also to draw conclusions extending the recent studies. In particular, escort histograms associated with MLE of image histograms obtained via the fixed–shift model [1], turned out to allow for a deeper insight into the origin of statistical difference of MLE of image histograms of medically treated and control samples. We attempted to balance unavoidable information loss due to using histograms instead of full images by quantifying the difference induced by medical treatment by the Hellinger distance between estimated histograms and identified conditions (given by image colors and type of sample staining) allowing for an inspection of histogram intensities responsible for the statistical difference. Shannon entropy of escort histograms, except its known properties [26], indicate non–uniform regions in an image due to recovery induced by applied medical treatment. In particular larger Shannon entropy of images of samples in femoral articular cartilage injury studied in Ref. [3] is a hallmark of the presence of clusters indicating effective recovery.

We conclude that the presented analysis causes us to believe that statistical image comparison can serve as a valuable tool for an auxiliary investigation of not–to–large sets of medical images. We claim that using statistical image analysis not only supports standard ‘human–based’ methods of selection of image parameters (such as color) or histological samples (such as applied staining) to represent and visualize the effectiveness of medical treatment but also can lead to new results with direct medical application and value.

## Supporting information

S1 Data (TXT)
